# Transgenic American Chestnuts Do Not Inhibit Germination of Native Seeds or Colonization of Mycorrhizal Fungi

**DOI:** 10.3389/fpls.2018.01046

**Published:** 2018-07-19

**Authors:** Andrew E. Newhouse, Allison D. Oakes, Hannah C. Pilkey, Hannah E. Roden, Thomas R. Horton, William A. Powell

**Affiliations:** Department of Environmental and Forest Biology, SUNY College of Environmental Science and Forestry, Syracuse, NY, United States

**Keywords:** risk assessment, allelopathy, ectomycorrhizae, leaf litter, chestnut blight, GMO, restoration

## Abstract

The American chestnut (*Castanea dentata*) was once an integral part of eastern United States deciduous forests, with many environmental, economic, and social values. This ended with the introduction of an invasive fungal pathogen that wiped out over three billion trees. Transgenic American chestnuts expressing a gene for oxalate oxidase successfully tolerate infections by this blight fungus, but potential non-target environmental effects should be evaluated before new restoration material is released. Two greenhouse bioassays evaluated belowground interactions between transgenic American chestnuts and neighboring organisms found in their native ecosystems. Potential allelopathy was tested by germinating several types of seeds, all native to American chestnut habitats, in the presence of chestnut leaf litter. Germination was not significantly different in terms of number of seeds germinated or total biomass of germinated seedlings in transgenic and non-transgenic leaf litter. Separately, ectomycorrhizal associations were observed on transgenic and non-transgenic American chestnut roots using field soil inoculum. Root tip colonization was consistently high (>90% colonization) on all plants and not significantly different between any tree types. These observations on mycorrhizal fungi complement previous studies performed on older transgenic lines which expressed oxalate oxidase at lower levels. Along with other environmental impact comparisons, these conclusions provide further evidence that transgenic American chestnuts are not functionally different with regard to ecosystem interactions than non-transgenic American chestnuts.

## Introduction

Transgenic American chestnuts (*Castanea dentata*) have been produced to express an oxalate oxidase enzyme (EC 1.2.3.4), which degrades toxic oxalic acid. Oxalic acid (or oxalate) is produced by the chestnut blight fungus (*Cryphonectria parasitica*) as a virulence factor that kills susceptible American chestnut cambium tissue. Degradation of this acid protects American chestnuts from the lethal effects of blight infections (Zhang B. et al., 2013; Newhouse et al., 2014) without harming the fungus. The oxalate oxidase used in transgenic chestnuts originated from wheat (Lane et al., 1993), but similar enzymes are found in many other monocots as well as unrelated taxa (Laker et al., 1980; Satyapal, 1993; Molla et al., 2013; NCBI Genbank, 2017), so the enzyme is not foreign to native ecosystems. Before any novel product is used for restoration, however, it is important to evaluate potential non-target environmental effects. This report describes two separate greenhouse bioassays to evaluate potential belowground impacts on other organisms found in American chestnut habitats. Chestnut leaf litter was observed for potential allelopathic effects on germination of native seeds, and chestnut roots were observed for potential effects on colonization by ectomycorrhizal fungi.

Bioassays to observe leaf effects on seed germination have been used to study plants with known allelopathic activity (Lodhi, 1978; Jäderlund et al., 1996; Yang et al., 2016), and to assess insect or microbial interactions with leaf litter from transgenic trees (Vauramo et al., 2006; Seppänen et al., 2007; Axelsson et al., 2011), but we are not aware of other published experiments specifically evaluating effects of transgenic tree leaf litter on seed germination. Given the relatively small number of forest-type deciduous trees transformed to date, it is not surprising that transgenic leaves have not been widely tested for potential effects on wild seed germination. However, assessments of environmental interactions are an important part of potential restoration projects, so such studies are prudent. Non-transgenic American chestnut leaves have previously been evaluated for allelopathic effects, and reduced germination has been reported on some seed species, but negligible effects were reported on other species (Good, 1968; Vandermast et al., 2002).

In nature, fine roots of most plants are colonized by fungi in a mutualistic symbiosis (Smith and Read, 2008). Plants provide their fungal partners with sugar, and fungi provide their plants phosphorus, nitrogen, and other mineral nutrients. It is generally accepted that the vast majority of land plants are normally mycorrhizal, and plants such as American chestnut require these fungi for normal growth. Given the importance of mycorrhizal fungi to American chestnut, it is particularly important to demonstrate that transgenic trees which can tolerate fungal infection above ground will still form partnerships with mutualistic fungi below ground. Transgenic American chestnuts have previously been shown to be no different than wild type Fagaceae with respect to colonization by ectomycorrhizal fungi in laboratory and field bioassays (Tourtellot, 2013; D’Amico et al., 2015). The current study examines newer transgenic lines with higher OxO expression than the ‘Darling 4’ line used in these older studies. The ‘Darling 54’ and ‘Darling 58’ lines used in both current studies express OxO mRNA at levels approximately similar to ‘Darling 215’ as described by Zhang B. et al. (2013).

## Materials and Methods

### Germination

All chestnut leaves (**Table 1**) were collected from a plot near Syracuse NY in the fall of 2016, dried at room temperature for approximately 6 months, and chopped to approximately 1 cm squares. Sixty grams of each leaf type was thoroughly mixed into 15 L of peat-based commercial potting mix (Fafard Germination Mix, Sungro Horticulture, Agawam, MA, United States), which had been moistened with 2 L of water. This mixture was evenly divided into three standard greenhouse seedling trays (25 cm × 51 cm × 6 cm) with drainage holes.

**Table 1:** Chestnut leaf types and names used in germination study.

**Chestnut type and details**	**Tree/line name (abbreviation)**
Wild-type (non-transgenic) American	‘McCabe Hollow’ (McCabe)
^∗^ Non-transgenic American parent line	‘Ellis 1’ (Ellis)
^∗^ Transgenic American (derived from Ellis parent line)	‘Darling 54’ (Dar 54)
^∗^ Transgenic American (derived from Ellis parent line)	‘Darling 58’ (Dar 58)
Chinese (*C. mollissima*)	‘Qing’
Hybrid (∼50% American, ∼50% Chinese)	F1 Hybrid (F1)
Third-generation Backcross (Steiner et al., 2017)	B3F3
No-leaf control	No Leaf

*^∗^Indicates trees of this type were used in the mycorrhizal colonization study.*

All seeds were purchased from Sheffield’s Seed Company (Locke, NY, United States) in the spring of 2017, and had been cold stratified by the supplier. Seed species were selected to represent different plant types of native species that are found in the traditional range and habitat of the American chestnut: *Elymus virginicus* = grass, *Cichorium intybus* = forb, *Gaultheria procumbens* = shrub, *Pinus strobus* = coniferous tree, *Acer rubrum* = deciduous tree. Twenty seeds of each species for each tray were started on March 21, 2017, either soaked in distilled water overnight (if suggested by the supplier for that species) or placed directly in the moist soil with leaves. Three seed types (*Tsuga canadensis, Cornus alterniflora, Tussilago farfara*, *n* = 10–15 seeds each) were sown in all trays but did not germinate appreciably in any tray type, so they were not included in subsequent analyses.

Transparent covers 7 cm tall were placed over all trays for the duration of the experiment, except during watering and observations. Trays were kept in a greenhouse at 20–22°C, with supplemental lighting on a 16-h light/8-h dark cycle. Trays were watered weekly; individual trays were watered more frequently if they were observed to be dry. Trays were arranged in three replicated blocks along a long table in the greenhouse, and left in place for the duration of the study.

**FIGURE 1:**
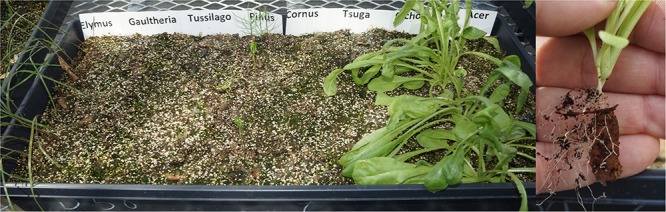
Representative tray to demonstrate germination bioassay in progress. This tray contained a single leaf type, and was one of three replicated trays with this leaf type. Note that *Cichorium* (second row from right) germinated notably earlier than other species, and was accordingly removed earlier to prevent crowding of adjacent rows. The smaller photo at right shows *Cichorium* roots growing through a chestnut leaf piece, demonstrating plant roots clearly interacting with leaf litter in this bioassay.

Germination observations, conducted twice weekly, consisted of counting the total number of seeds that had germinated of each type in each tray (**Figure 1**). At the conclusion of observations for a given species, all germinated seedlings were removed from the tray, tapped and brushed gently to remove loose potting mix, dried at 60°C in a paper bag for 48 h, and total seedling dry biomass was recorded for each species in each tray. (This was conducted at ∼4 weeks for *Cichorium*, of which essentially all seeds had already germinated and many were crowding seedlings in adjacent rows, and 8–10 weeks for remaining species.) Mean counts and masses from each tray were analyzed with one-way ANOVA (GLM Procedure, SAS v9.2, SAS Institute, Cary, NC, United States) and compared using Tukey’s Studentized Range (HSD) test (α = 0.05).

### Mycorrhizal Colonization

Soil samples for mycorrhizal inoculum were collected from the same plot as the leaves, in mixed hardwood forest with sugar maple (*Acer saccharum*), American beech (*Fagus grandifolia*) and Eastern hemlock (*Tsuga canadensis*). Twenty-three samples were taken using a cylindrical soil core 4 cm diameter driven to a depth of 15 cm. The location of each sample was randomly determined. Soil samples were dried, then sifted through a 0.5 cm mesh (USA Standard Soil Sieve). The soil inoculant was mixed at a ratio of 1:1:2 dried soil: sand: sphagnum peat moss. The resulting inoculant mix was split evenly among 45 pots (D40 Deepots, Stuewe & Sons), which had been previously sterilized overnight in a 7% bleach solution. Tissue culture-generated *C. dentata* approximately 6 months old were transplanted into pots containing the inoculant. Three types of *C. dentata* were used: 15 individuals each of ‘Ellis 1,’ ‘Darling 54,’ and ‘Darling 58’ (**Table 1**). These were grown a greenhouse at 21–26°C, with a 16-h light/8-h dark cycle, and watered as needed. The plants did not receive fertilizer or pH amendments during the experiment to encourage associations with mycorrhizal fungi.

**FIGURE 2:**
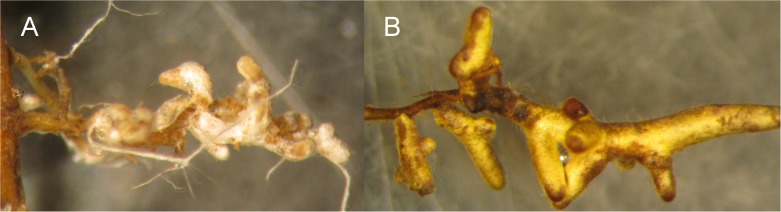
Ectomycorrhizal (EM) root tips harvested from American chestnut seedlings. Plant roots are seen on the left side of both figures as a brownish main (secondary) root. EM roots branch off of these secondary roots and are wrapped in a fungal sheath or mantle. The fungal mantle gives the EM root tips their distinctive colors, with a white morphological type (morphotype) in **(A)** and a golden morphotype in **(B)**. Note the hyphal cords in **(A)**: these are aggregations of hyphae that facilitate transport of sugars from the plant to the fungus in the soil and soil nutrients from the fungus to the plant roots.

Mycorrhizal colonization rate was assessed after 5 months of growth by collecting a continuous root length of at least 15 cm from each surviving plant. All root tips along the sample were observed, and the percentage of root tips with evidence of a fungal mantle, and those without a mantle, were visually estimated using a dissecting microscope and assigned to categorical percentage ranks (e.g., 90–95 or >95%). A root tip was considered ectomycorrhizal if it was actively colonized or senescent with indications that it had been previously colonized. Ectomycorrhizal roots are produced when a mycorrhizal fungus forms a mantle around root tips. Evidence of colonization is readily apparent on chestnut roots (**Figure 2**); fungal mantles are distinctly unique in terms of color, texture, and thickness compared to un-colonized areas of roots. Non-mycorrhizal roots were identified by the lack of a mantle and presence of root hairs. The frequencies of each category in each treatment were calculated. A Fisher’s exact test of independence with a significance of 0.05 was used to test the null hypothesis that there was no significant difference in root tip colonization between any two treatments.

## Results

### Germination

**FIGURE 3:**
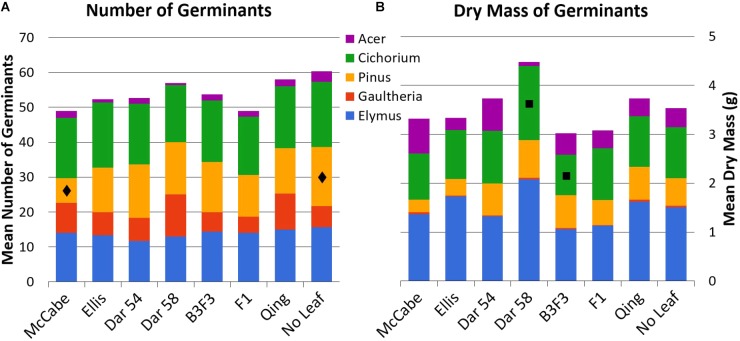
Total mean counts (**A**, *n* = 20 seeds/type) and dry masses **(B)** of germinated seeds for all leaf and seed types. All mean values with standard error are available as **Supplementary Material**. See **Table 1** for leaf types and abbreviations. Statistically significant differences for pairwise comparisons within seed species are noted with 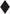 (*Pinus* counts, McCabe vs. No Leaf) and 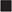 (*Cichorium* mass, ‘Darling 58’ vs. B3F3).

Mean counts and masses of germinated seedlings in all leaf types are shown in **Figure 3**; all mean values with standard error are available as **Supplementary Material**. Tukey’s HSD test indicated only a few pairwise comparisons with statistically significant (*p* < 0.05) differences (presented here as mean ± 1 SE). Count of *Pinus* seedlings was significantly different between ‘McCabe Hollow’ (7.0 ± 1.2 germinants) and the no-leaf control (17.0 ± 2.5 germinants), and the mean biomass of *Cichorium* seedlings was significantly different between ‘Darling 58’ (1.52 ± 0.19 g) and B3F3 (0.83 ± 0.14 g). There were no significant differences between either of the transgenic leaf types and the non-transgenic ‘Ellis 1’ control, which is genetically identical to the ‘Darling’ lines in this experiment other than transgene presence. Allelopathy by chestnut leaves in general was not broadly apparent, as no-leaf control trays showed overall similar germination of most seed species.

### Mycorrhizal Colonization

Surviving trees observed in the mycorrhizal colonization study included 15 ‘Ellis,’ 10 ‘Darling 54,’ and 12 ‘Darling 58.’ Mycorrhizal colonization was consistently high among all types, with all but one observed plant showing greater than 95% ectomycorrhizal colonization. The observed root from one ‘Darling 54’ tree showed 90–95% colonization. According to Fisher’s exact tests, there were no significant differences in colonization between ‘Ellis’ and the transgenic lines ‘Darling 54’ or ‘Darling 58’ (*p* > 0.40).

## Discussion

The few statistically significant differences in seedling germination between leaf types (*Pinus* counts, *Cichorium* mass) did not represent trends between transgenic and non-transgenic American chestnuts. In both of those contrasts, similar leaf types (**Table 1**) did not show the same patterns. In other words, while the *Pinus* germination count was low on ‘McCabe Hollow’ leaves, it was not significantly different among other American chestnut leaf types. And while *Cichorium* mass was higher on ‘Darling 58’ leaves than B3F3 leaves, this difference was not statistically significant compared to American chestnut controls or other leaf types. These differences may therefore be due to individual genotypic differences or random variability, and are within the scope of differences found in other non-transgenic controls in this study. The general lack of germination observed in the three excluded seed species (*Tsuga canadensis, Cornus alterniflora, Tussilago farfara*) may indicate inadequate cold stratification, but its consistency among all leaf types and no-leaf controls suggests it is not the result of leaf interactions or allelopathy.

Previous studies on transgenic leaf litter have more commonly focused on microbial communities or leaf decomposition rates rather than germination of neighboring plant seeds. Not surprisingly, some transgene products in such studies have been observed to produce their intended effect when present in leaf litter: Bt toxins in leaf litter can affect aquatic insect communities (Axelsson et al., 2011) and increased tannin levels in leaf litter can affect moss proliferation and certain microbial classes (Winder et al., 2013). In both of these cases, the changes observed are expected based on the transgene product, and other microbial communities were not affected. The oxalate oxidase enzyme in ‘Darling’ transgenic American chestnuts is not a toxin and is not known to have allelopathic properties [it actually degrades toxic oxalate which likely *does* have allelopathic properties (Itani et al., 1999)], so the germination similarities among chestnut leaf types are not surprising.

Colonization by mycorrhizal fungi is often one of the first concerns people express when they hear about a wild, non-agricultural plant engineered to tolerate infections by a pathogenic fungus. Such concerns are of course legitimate, especially in cases where broad-spectrum fungicidal traits might be employed. But in the case of ‘Darling’ chestnuts, the transgene product is not fungicidal in nature, and did not affect mycorrhizal colonization. This study specifically supports previous investigations on the mycorrhizal condition of transgenic American chestnut based on field and laboratory bioassays, all of which indicate no differences in colonization between transgenic and non-transgenic American chestnut roots (Tourtellot, 2013; Dulmer et al., 2014; D’Amico et al., 2015). Even with the higher transgene expression in ‘Darling 54’ and ‘Darling 58’ compared to the ‘Darling 4’ tested previously, there were no significant differences in colonization by ectomycorrhizal fungi in roots compared to non-transgenic controls. These results corroborate other studies that generally show no significant differences between transgenic and non-transgenic plants with respect to colonization of mycorrhizal fungi (Kaldorf et al., 2002; Newhouse et al., 2007; Cheeke et al., 2015; Turrini et al., 2015; Chen et al., 2016; Kaur et al., 2017).

Instead of acting directly on the pathogen, the transgene product (oxalate oxidase) in ‘Darling 54’ and ‘Darling 58’ chestnuts protects the host by degrading a toxin produced by the chestnut blight fungus. The toxin, oxalic acid, is associated with wood decay in some fungal species (Clausen and Green, 2003; Hastrup et al., 2012), and in chestnut blight, it is a virulence factor specifically associated with the pathogenic lifestyle of *C. parasitica* (Rigling and Prospero, 2018). In contrast, mycorrhizal fungi depend on the mutual flow of materials between themselves and their plant hosts, and thus have no need for the action of oxalic acid, so its degradation should have no effect on mycorrhizal colonization. Beyond the transgene product itself, the by-products from oxalate oxidase degradation of oxalic acid are hydrogen peroxide and carbon dioxide (Lane et al., 1993). Hydrogen peroxide has fungicidal properties at sufficiently high concentrations (Baldry, 1983; El-Gazzar and Marth, 1988), but in some cases mycorrhizal fungi may actually employ hydrogen peroxide as a control mechanism (Salzer et al., 1999) or signal molecule (Zhang R. et al., 2013). Furthermore, chestnut blight does not typically infect tree roots (Hepting, 1974; Weidlich, 1978), so it is unlikely that substantial oxalic acid degradation (and thus hydrogen peroxide formation) would take place in the rhizosphere.

Collectively, these studies reinforce previous and concurrent findings that transgenic American chestnuts are not ecologically different than non-transgenic American chestnuts (apart from their enhanced blight tolerance). Along with the previous mycorrhizal experiments referenced above, collaborators have preliminarily evaluated aquatic and terrestrial insect feeding on transgenic chestnut leaves and natural introgression of plants near field-planted transgenic chestnut trees (unpublished). Additionally, Gray (2015) and Gray and Briggs (2015) compared transgenic and non-transgenic chestnut leaf decomposition, and Goldspiel et al. (in press) tested wood frog tadpole growth and survival with transgenic chestnut leaves. In each of these cases, transgenic chestnuts showed negligible differences compared to non-transgenic American chestnuts, or smaller differences than traditionally-bred hybrid or Chinese chestnuts. Restoration of wild species such as American chestnut should be approached with care and wisdom regardless of what methods are used to produce restoration material, and interactions with neighboring species are an important part of this biosafety evaluation process.

## Data Availability

The raw data supporting the conclusions of this manuscript will be made available by the authors, without undue reservation, to any qualified researcher.

## Author Contributions

AN wrote the majority of the manuscript and helped with setup, observations, and analysis of the germination study. AO helped with design and setup of the germination study and performed statistical analyses for this experiment. HP helped with setup and performed the majority of observations for the germination study. HR helped with setup and performed all observations and statistical analysis for the mycorrhizae study. TH contributed advice on the mycorrhizal experiment and helped with the writing of this part of the manuscript. WP was the chestnut project director and provided lab space, greenhouse space, transgenic plant material for both studies, and valuable support and experimental guidance. All authors reviewed and approved the manuscript prior to submission.

## Conflict of Interest Statement

The authors declare that the research was conducted in the absence of any commercial or financial relationships that could be construed as a potential conflict of interest.

## Supplementary Material

DATA SHEET S1 Mean seedling counts and dry biomass measurements (both including standard error) for germinated seedlings with all leaf types.
